# Optimized Long‐TE ^1^H sLASER MR Spectroscopic Imaging at 3T for Separate Quantification of Glutamate and Glutamine in Glioma

**DOI:** 10.1002/jmri.29787

**Published:** 2025-04-08

**Authors:** Seyma Alcicek, Michael W. Ronellenfitsch, Joachim P. Steinbach, Andrei Manzhurtsev, Dennis C. Thomas, Katharina J. Weber, Vincent Prinz, Marie‐Thérèse Forster, Elke Hattingen, Ulrich Pilatus, Katharina J. Wenger

**Affiliations:** ^1^ Goethe University Frankfurt, University Hospital, Institute of Neuroradiology Frankfurt am Main Germany; ^2^ University Cancer Center Frankfurt (UCT) Frankfurt am Main Germany; ^3^ LOEWE Frankfurt Cancer Institute (FCI) Frankfurt am Main Germany; ^4^ German Cancer Research Center (DKFZ) Heidelberg Germany and German Cancer Consortium (DKTK) Frankfurt am Main Germany; ^5^ Goethe University Frankfurt, University Hospital, Dr. Senckenberg Institute of Neurooncology Frankfurt am Main Germany; ^6^ Goethe University Frankfurt, University Hospital, Institute of Neurology (Edinger‐Institute) Frankfurt am Main Germany; ^7^ Goethe University Frankfurt, University Hospital, Department of Neurosurgery Frankfurt am Main Germany

**Keywords:** ^1^H sLASER long‐TE, brain tumor, glutamate, glutamine, MR spectroscopy, reproducibility

## Abstract

**Background:**

Glutamate and glutamine are critical metabolites in gliomas, each serving distinct roles in tumor biology. Separate quantification of these metabolites using in vivo MR spectroscopy (MRS) at clinical field strengths (≤ 3T) is hindered by their molecular similarity, resulting in overlapping, hence indistinguishable, spectral peaks.

**Purpose:**

To develop an MRS imaging (MRSI) protocol to map glutamate and glutamine separately at 3T within clinically feasible time, using J‐modulation to enhance spectral differentiation, demonstrate its reliability/reproducibility, and quantify the metabolites in glioma subregions.

**Study Type:**

Prospective.

**Population:**

Phantoms, 5 healthy subjects, and 30 patients with suspected glioma. IDH wild‐type glioblastoma cases were evaluated to establish a uniform group.

**Field Strength/Sequence:**

3T, Research protocol: 2D ^1^H sLASER MRSI (40 and 120 ms TE) with water reference, 3D T1‐weighted and 2D T2‐weighted. Trial‐screening process: T1‐weighted, T1‐weighted contrast‐enhanced, T2‐weighted, FLAIR.

**Assessment:**

Spectral simulations and phantom measurements were performed to design and validate the protocol. Spectral quality/fitting parameters for scan‐rescan measurements were obtained using LCModel. The proposed long‐TE data were compared with short‐TE data. BraTS Toolkit was employed for fully automated tumor segmentation.

**Statistical Tests:**

Scan‐rescan comparison was performed using Bland–Altman analysis. LCModel coefficient of modeling covariance (CMC) between glutamate and glutamine was mapped to evaluate their model interactions for each spectral fitting. Metabolite levels in tumor subregions were compared using one‐way ANOVA and Kruskal‐Wallis. A *p* value < 0.05 was considered statistically significant.

**Results:**

Spectral quality/fitting parameters and metabolite levels were highly consistent between scan‐rescan measurements. A negative association between glutamate and glutamine models at short TE (CMC = −0.16 ± 0.06) was eliminated at long TE (0.01 ± 0.05). Low glutamate in tumor subregions (non‐enhancing‐tumor‐core: 5.35 ± 4.45 mM, surrounding‐non‐enhancing‐FLAIR‐hyperintensity: 7.39 ± 2.62 mM, and enhancing‐tumor: 7.60 ± 4.16 mM) was found compared to contralateral (10.84 ± 2.94 mM), whereas glutamine was higher in surrounding‐non‐enhancing‐FLAIR‐hyperintensity (9.17 ± 6.84 mM) and enhancing‐tumor (7.20 ± 4.42 mM), but not in non‐enhancing‐tumor‐core (4.92 ± 3.38 mM, *p* = 0.18) compared to contralateral (2.94 ± 1.35 mM).

**Data Conclusion:**

The proposed MRSI protocol (~12 min) enables separate mapping of glutamate and glutamine reliably along with other MRS‐detectable standard metabolites in glioma subregions at 3T.

**Evidence Level:**

1

**Technical Efficacy:**

Stage 3

**Plain Language Summary**

- Gliomas, the most common primary brain tumors, show a heterogeneous metabolism.
- Glutamate and glutamine play distinct roles in glioma growth, making their non‐invasive detection clinically highly attractive.
- The challenge is to report both metabolite levels separately, as their signals overlap in conventional MR spectroscopic imaging (MRSI) at clinical field strengths.
- To overcome this challenge, a dedicated MRSI protocol was developed, and its reproducibility/reliability was tested.
- With this protocol, regional differences in glutamate and glutamine concentrations for the most aggressive glioma subtype were demonstrated.
- This research furthers the ability to study tumor metabolism in vivo and may help guide metabolism‐targeted treatments.

## 1 Introduction

Adult‐type diffuse gliomas are a heterogeneous group of brain tumors [1]. Metabolic reprogramming in the tumor microenvironment is a hallmark of glioma [2]. In particular, somatic mutations in the genes encoding the enzymes isocitrate dehydrogenase 1 and 2 (IDH1/2) alter tumor metabolism, leading to distinct biological behaviors [3]. As a result, the presence of an IDH mutation is an important biomarker for patient symptomology, prognosis, and glioma classification [1]. Patients with IDH‐mutant (IDHmut) gliomas experience a higher frequency of seizures compared to those with IDH wild‐type (IDHwt) [4]. Furthermore, IDHwt gliomas are associated with shorter overall survival and poorer treatment response [5].

One of the key metabolites in diffuse glioma, promoting glioma invasion and growth, is glutamate (Glu) which serves as a neurotransmitter as well as an energy source and building block for biomolecules [6]. Glioma cells primarily secrete Glu via the cystine/Glu antiporter solute carrier family 7 member 11 (SLC7A11 or xCT) [7]. Overexpression of xCT (SLC7A11) has been hypothesized to play an important role in the increase of ambient non‐synaptic Glu levels and the development of a hyperexcitable peritumoral network leading to glioma‐associated epileptic discharges and excitotoxicity [8]. High xCT expression has been proposed as a biomarker for glioma‐related epilepsy, independent of IDH mutation status, with IDHwt tumors exhibiting higher xCT levels than their mutated counterparts [9]. Other potential mechanisms for extracellular Glu accumulation are overexpression of branched‐chain amino acid transaminase 1 (BCAT1), which produces Glu through transamination, and impairment of sodium‐dependent re‐uptake of Glu in glioma tissue due to a downregulated expression or mislocalization of the Glu transporter 1 (GLT1) [10, 11]. The major substrate for the production of Glu is glutamine (Gln). Gln is a nitrogen reservoir and an energy source for tumor cells. High Gln levels are associated with tumor growth and treatment resistance [12, 13].

Understanding alterations in glutamatergic tumor metabolism is needed for developing targeted metabolic therapies and identifying biomarkers for diagnosis and treatment monitoring [12, 14]. The changes in the level of key metabolites in glutamatergic mechanisms (i.e., Glu and Gln) can be measured non‐invasively in the human brain using in vivo proton magnetic resonance spectroscopy (^1^H‐MRS) [15], as they are relatively abundant amino acids with levels of 6–13 mmol and 3–6 mmol per kg brain tissue, respectively [16, 17].

However, the similarity of their molecular structures results in hardly distinguishable, overlapping spectral patterns, hindering their separate quantification [15]. Therefore, the sum of Glu and Gln, often referred to as Glx in the literature, has been reported in in vivo MR spectroscopy studies performed at clinical field strengths (≤ 3 T), despite their distinct roles in the healthy human brain as well as in tumor metabolism, hindering precise interpretation.

In order to quantify Glu and Gln simultaneously and separately, standard spectroscopic sequences (PRESS, Point RESolved Spectroscopy, and STEAM, STimulated Echo Acquisition Mode) have been optimized employing different echo times (TE) [18, 19]. However, using the semi‐adiabatic localization by adiabatic selective refocusing (sLASER) sequence has been recommended over these sequences [20]. The sLASER localization scheme causes lower chemical shift displacement error, suppresses anomalous J‐evolution, and reduces sensitivity to B_1_^+^ inhomogeneity.

Furthermore, many previous ^1^H MRS studies at clinical field strengths investigating glutamatergic mechanisms in glioma have relied on single‐voxel measurements. Although the discrimination of Glu and Gln benefits from the improved spectra quality, the diagnostic value of these methods is limited by poor spatial resolution [21, 22, 23, 24]. Since metabolite levels may differ between tumoral and peritumoral subregions (e.g., solid tumor, necrosis, and peritumoral edema), single‐voxel MRS measurements are subject to partial volume effects [25], which may contribute to inconsistencies in reported metabolite levels across tumor types.

This study aimed to obtain distinct spectral patterns of Glu and Gln by taking advantage of J‐modulation at an optimized TE at 3 T for the vendor‐provided ^1^H sLASER MRS imaging (MRSI) sequence, establish a protocol to identify specific alterations in Glu and Gln concentrations in a clinically feasible scan time (~12 min), and finally apply it to a cohort of patients with IDHwt glioblastoma for quantifying/mapping Glu and Gln as separate signals within tumoral and peritumoral subregions.

## 2 Materials and Methods

### 2.1 Spectral Simulations and Phantom Experiments

^1^H spectra for metabolites of interest were simulated using the jMRUI plug‐in NMR‐ScopeB [26] (Version 6.0, available at http://www.mrui.uab.es), with prior knowledge of chemical shifts and *J‐*coupling constants [15, 27]. Echo times ranging from 40 to 144 ms with sequence timings and refocusing RF pulses as applied in sLASER were used, assuming ideal excitation. The simulated spectra were used as basis sets for spectral fitting of in vitro and in vivo measurements. T_2_ decay was neglected in the simulations. 2‐hydroxyglutarate (2HG), an oncometabolite that accumulates in IDHmut gliomas [28], was included in the simulation to evaluate spectral overlaps with Glu and Gln.

A phantom containing phosphate‐buffered saline with 20 mM Glu, 20 mM Gln, and 10 mM creatine (Cr) (pH = 7.1) was prepared (phantom‐1). The phantom was measured on a clinical whole‐body 3T MR Scanner (MAGNETOM Prisma (VE11C), Siemens Healthineers; Erlangen, Germany) using a vendor‐supplied 20‐channel ^1^H head coil. ^1^H spectra of the phantom were acquired with the vendor‐provided sLASER pulse sequence with TEs of 100–140 ms, with 10 ms increments (see Table S1 for the other sequence parameters).

In addition, a brain‐mimicking phantom (phantom‐2) containing 10 mM Cr, 10 mM Glu, 5 mM Gln, 3 mM choline (Cho), 3 mM glutathione (GSH), 2 mM γ‐aminobutyric acid (GABA), 7.5 mM myo‐inositol (mI), 5 mM lactate (Lac), and 12.5 mM N‐acetylaspartate (NAA) in phosphate‐buffered saline (pH = 7.2) was prepared. The phantom was measured with a conventional short‐TE (40 ms) and the proposed long‐TE (120 ms). Quantitation based on Quantum Estimation (QUEST) [29] in jMRUI was employed for time‐domain spectral fitting with simulated basis set and residual spectra calculation. Cr was used as a reference metabolite.

### 2.2 Clinical Study Design

**FIGURE 1:**
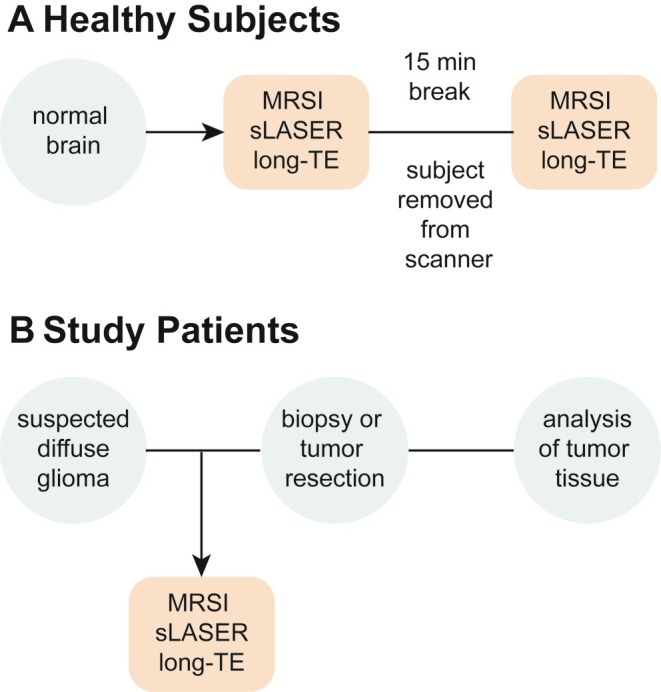
Study workflow for (A) healthy subjects and (B) patients with diffuse glioma. ^1^H MRSI protocol was repeated once for healthy subjects to analyze scan‐rescan reproducibility.

The prospective study was conducted at a tertiary care hospital. The study protocol was approved by the local institutional review board (Project Number 2022–909) and written informed consent was obtained from all participants. The study was registered in the German Clinical Trials Register (DRKS; No 00032097). To evaluate the reliability and repeatability of the protocol, five healthy subjects were enrolled, scanned, and evaluated (Figure 1A). Upon protocol validation, 30 patients (≧ 18 years of age) with MRI‐suspected diffuse glioma according to the 2021 World Health Organization Central Nervous System (WHO CNS) tumor classification [1] and a recommendation for biopsy/resection were recruited (Figure 1B). The MRS measurement was followed by tumor biopsy/resection. Only patients diagnosed with IDHwt glioma were included in the analysis to create a homogenous cohort. Electronic medical records were analyzed for demographic information and tumor characteristics.

### 2.3 Study Protocols

Five healthy subjects were scanned in two separate sessions using an MRS protocol on a clinical whole‐body 3T MR Scanner (MAGNETOM Prisma (VE11C), Siemens Healthineers, Erlangen, Germany) with a vendor‐supplied 20‐channel ^1^H head coil. The volume of interest (VOI) was placed in the subjects' medial fronto‐parietal lobes. Each subject completed two sessions with a short break (~15 min). In between the sessions, the subject was removed from the scanner. The protocol included the 2D ^1^H MRSI sLASER sequence with the proposed long‐TE (120 ms), 3D T1‐weighted (T1W), and 2D T2‐weighted (T2W) MRI for reference images. One healthy subject was measured additionally with a conventional short‐TE (40 ms) sLASER MRSI for comparison.

All enrolled patients had undergone routine brain tumor imaging as part of the trial screening process, with T1W, contrast‐enhanced T1W, T2W, and FLAIR images available for analysis. In the MRSI study, a transversal slice was positioned to cover solid tumor tissue and contralateral normal‐appearing brain tissue. The VOI was selected by a combination of the sLASER and outer volume suppression, enclosing the tumor and the respective area in the contralateral hemisphere. For absolute quantification of metabolite concentrations in the tumor, the same sequence with lower resolution and without water suppression was recorded for an identical VOI. Table S1 provides the detailed MRS sequence protocol. In addition, the MRS in MRS Reporting Checklist can be found in Table S2.

### 2.4 Data Processing and Metabolite Quantification

Registration of the multimodal spectroscopic data to 3D‐anatomical data was performed with an in‐house software tool, which was scripted in MATLAB (The Mathworks Inc., Natick; Massachusetts, USA). A graphical user interface implemented in this tool allowed the selection of voxels from the entire spectroscopic dataset using 3D‐T1W and 2D‐T2W reference images with an MRSI grid overlay. The quality of ^1^H MRS data was evaluated with the following rejection criteria: Metabolite linewidth (FWHM) > 0.1 ppm, existing artifacts, and the highest metabolite signal‐to‐noise ratio < 3.

LCModel [30] was used for the ^1^H spectral analysis. Metabolite signals for the basis set were simulated as explained above for the sLASER pulse sequence at TE of 40 and 120 ms. The basis set included spectra of NAA, N‐acetylaspartylglutamate (NAAG), Cho, glycerophosphocholine (GPC), Cr, Glu, Gln, mI, Lac, GABA, GSH, glycine (Gly), alanine (Ala), glucose (Glc), and valine (Val). As shown in the study by Gajdošík M et al. [31], baseline signals with a short T_2_ relaxation time (average of 21.9 ms) decay to < 0.5% at long TE (120 ms). Therefore, expecting low signals from macromolecules and lipids, a rigid baseline modeling was applied. This was accomplished by adjusting the LCModel parameter that controls knot spacing (DKNTMN) to 5 ppm. For the remaining macromolecule and lipid signals, the LCModel default basis set of macromolecule and lipid resonances was used.

After LCModel analysis, the phased, fitted spectra as well as the residuals were evaluated visually to check the quality of the fit. Spectral fitting quality was evaluated with a rejection threshold using Cramer‐Rao lower bounds (CRLB) of metabolite fits [32] as < 10% for tCho (total choline) due to expected variations of metabolite levels in tumor subregions. Metabolite ratios were calculated for the MRSI study on healthy subjects. Reproducibility and reliability analyses were performed using spectra from all voxels, excluding those at the edge of the VOI due to imperfect slice profiles, resulting in 100 spectra per measurement.

For ^1^H MRSI data acquired from glioma patients, metabolite quantification was performed using the tissue water content detected by the low‐resolution 2D ^1^H sLASER MRSI pulse sequence without water suppression. Metabolite concentrations in tissue (mol per tissue volume) were calculated according to:

(1) $$C_{\mathrm{met}}\left(i,j\right)=\frac{S_{\mathrm{met}}\left(i,j\right)\times N_{W}}{S_{W}\left(i,j\right)\times N_{\mathrm{met}}}\times C_{W}\left(i,j\right)$$

where $C_{\mathrm{met}}\left(i,j\right)$ and $C_{W}\left(i,j\right)$ represent the absolute concentrations of metabolite and water in the voxel $\left(i,j\right),$ respectively; $S_{\mathrm{met}}\left(i,j\right)$ and $S_{W}\left(i,j\right)$ refer to the signals of metabolite and water in the respective volume; $N_{W}$ and $N_{\mathrm{met}}$ refer to the number of protons in water and metabolite. With this approach, B_1_ (receive and transmit) effects are canceled, as the same sequence with the same VOI is used for measuring the metabolite and water. For contralateral normal‐appearing tissue, $C_{W}$ were calculated based on tissue composition (white matter, gray matter, cerebrospinal fluid) determined from segmented data and literature values of water content in each tissue type. $C_{W}\left(i,j\right)$ obtained from segmented data was adjusted by digital filtering to match the poor point spread function (PSF) of $S_{W}\left(i,j\right)$. Following the calculation of reliable absolute metabolite concentration in contralateral normal‐appearing tissue ($C_{\mathrm{met}}\left({\mathrm{CL}}_{\mathrm{ref}}\right)$), this concentration was used as a reference for the tumor area by applying:

(2) $$C_{\mathrm{met}}\left(i,j\right)=\frac{S_{\mathrm{met}}\left(i,j\right)}{S_{\mathrm{met}}\left({\mathrm{CL}}_{\mathrm{ref}}\right)}\times C_{\mathrm{met}}\left({\mathrm{CL}}_{\mathrm{ref}}\right)$$

where $S_{\mathrm{met}}\left({\mathrm{CL}}_{\mathrm{ref}}\right)$ refers to the metabolite signals in contralateral normal‐appearing tissue, with the assumption that the vendor‐supplied receive field inhomogeneity correction routine, “prescan normalize” effectively corrects for $B_{1}^{-}\left(i,j\right)$ (receiver field) inhomogeneities and the adiabatic pulses in the sLASER sequence are inherently immune to $B_{1}^{+}\left(i,j\right)$ (transmit field) variations. The procedure is detailed in the Supporting Information including corrections for T_1_ and T_2_ relaxation.

The BraTS Toolkit [33] was used for fully automated segmentation of glioma subregions on co‐registered routine brain tumor imaging data from the trial screening process. The tumor volume was segmented into three classes: Surrounding non‐enhancing FLAIR hyperintensity (SNFH), non‐enhancing tumor core (NETC), and enhancing tumor (ET) [34]. For two cases, manual segmentation had to be performed due to missing sequences for fully automated segmentation. The segmentations were visually verified by a neuroradiology specialist (K.J. Wenger) with 8 years of experience.

Tumor segmentations were co‐registered to the MRSI space using FSL Linear Registration Tools (FLIRT) [35] with the anatomical reference images, that is T1W images obtained in each image space. The fraction of each tumor subregion in each voxel was calculated using the in‐house software tool. Voxels with more than 50% from a specific subregion were assigned to this subregion and ordered according to the fraction of the respective subregion. Metabolite levels were calculated from the three voxels with a maximum fraction. When the fraction in the three voxels was the same as the subsequent order voxels, they were also included in the analysis. When there was no voxel with at least > 50% of the respective tumor subregion, the dataset was not included in the statistical analysis for the respective tumor subregion. In addition, SPM12 (version 7771) was used for segmentation of normal‐appearing brain tissue [36]. The segmented data were used for water quantification as explained above and in the Supporting Information.

### 2.5 Statistical Analysis

Statistical analysis was performed using R v4.4.1 in RStudio v2024.04.2, except for the Bland–Altman analysis. The mean and standard deviation of spectral quality and fitting parameters (i.e., FWHM, SNR, and CRLBs) were calculated. Bland–Altman bias (the mean difference) between sessions was computed for the Glu/tNAA and Gln/tNAA ratios using OriginPro software (version 2020; OriginLab Corp., Northampton, MA, USA). LCModel coefficients of modeling covariance (CMC) were derived from the Fisher information matrix, representing the interaction between two model parameters within a single spectrum. These coefficients were used to evaluate the distinguishability of Glu and Gln in a spectrum acquired at the conventional short‐TE (40 ms) and the proposed long‐TE values (120 ms). Glu and Gln concentrations were compared across tumor subregions for IDHwt glioma. The normality of metabolite distribution within each tumor subregion was assessed using the Shapiro–Wilk test. For normally distributed data, a one‐way analysis of variance was applied, followed by Tukey's honest significant difference post hoc test to identify significant pairwise differences between subregions. For non‐normally distributed data, the Kruskal‐Wallis test was used, followed by Dunn's test with Benjamini‐Hochberg correction for multiple comparisons. Results were considered significant at *p* < 0.05.

## 3 Results

### 3.1 Spectral Simulations and Phantom Study for ^1^H MRSI Protocol Optimization

**FIGURE 2:**
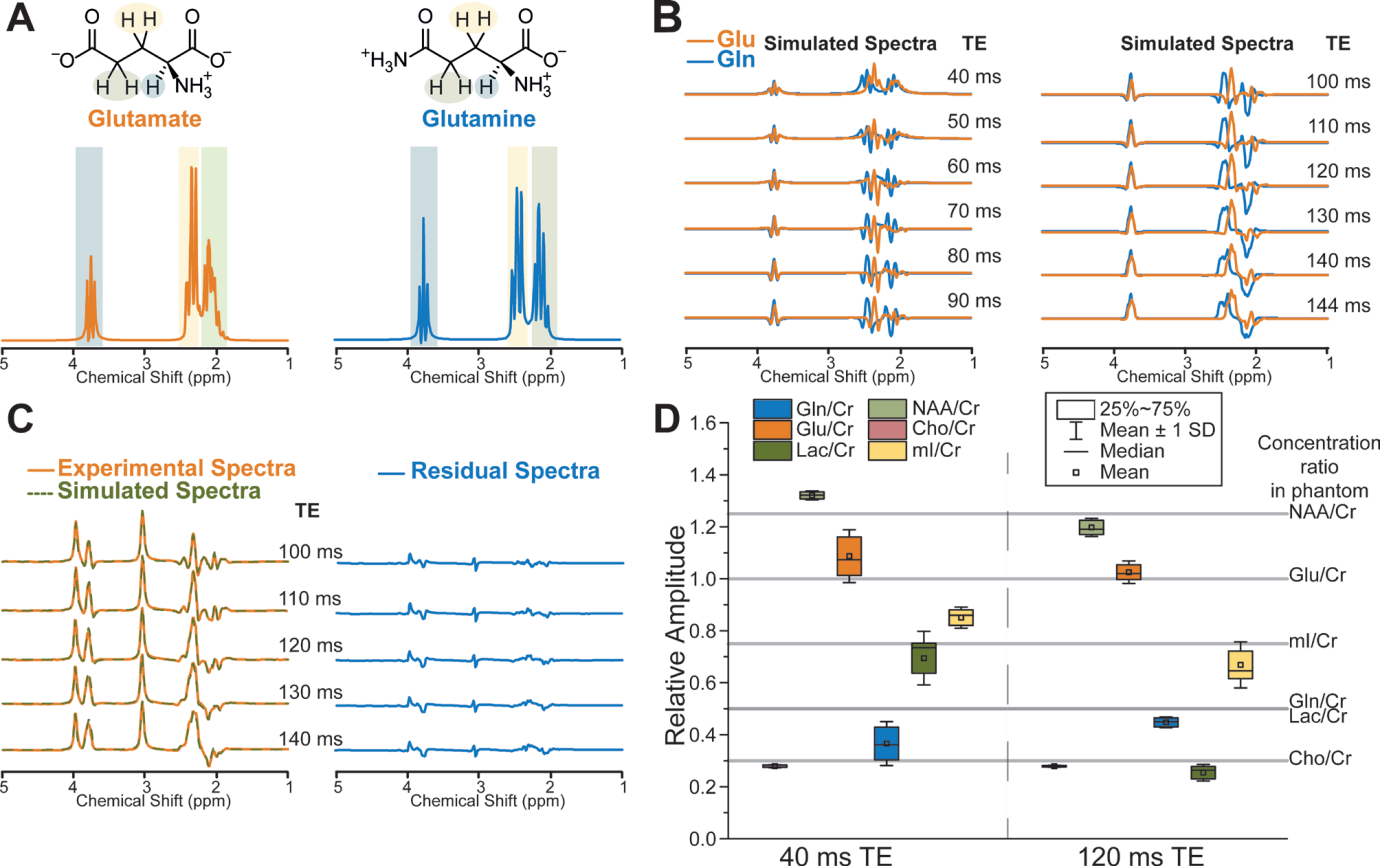
(A) Molecular structure and simulated ^1^H spectra of glutamate (Glu) and glutamine (Gln) (TE = 30 ms). (B) Simulated spectra of Glu and Gln at various echo times (TE) for ^1^H MR spectroscopic imaging (MRSI) using sLASER sequence. (C) Experimental (phantom‐1) and superimposed simulated spectra of Glu, Gln, and creatine (Cr) at various TE (100–140 ms) together with residual spectra. (D) The relative amplitudes of metabolites calculated for multiple MRSI voxels' spectra acquired from the brain‐tumor‐mimicking phantom (phantom‐2) using sLASER sequence at TE of 40 and 120 ms. T_1_ and T_2_ relaxation corrections were not applied.

Simulated spectral patterns of Glu and Gln as a function of TE (40–144 ms) in sLASER are demonstrated in Figure 2B. Spectral patterns of J‐coupled spin metabolites varied with changing TEs due to the J‐evolution of the coupled resonances. At a TE of 120 ms, the peaks in the Glu spectrum are mostly in phase, whereas the spectral pattern originating from Gln consists of in‐phase and anti‐phase components, which may facilitate separate quantification of Glu and Gln. In addition, no prominent 2HG peak overlaps with the anti‐phase component of the Gln spectrum, as shown in Figure S3.

The accuracy of spectral simulations in replicating spectral patterns influenced by J‐evolution at long TE was validated using phantom measurements. The spectral fitting of data acquired from phantom‐1 containing 20 mM Glu, 20 mM Gln, and 10 mM Cr using sLASER MRSI with TEs of 100–140 ms is shown in Figure 2C. A good visual agreement between phantom measurements and simulations was observed with the minimal residual spectra proving the validity of numerical simulations for the respective protocol.

^1^H MRSI data obtained from a brain‐mimicking phantom (phantom‐2) demonstrated lower variability (i.e., standard deviation) in the quantification of Glu/Cr (120 ms: 1.03 ± 0.04 and 40 ms: 1.09 ± 0.1), Gln/Cr (0.45 ± 0.02, 0.37 ± 0.08), Cho/Cr (0.28 ± 0.003, 0.27 ± 0.006), and Lac/Cr (0.25 ± 0.03, 0.69 ± 0.1) at a TE of 120 ms compared to 40 ms (Figure 2D). In contrast, higher variability was observed in the quantification of mI/Cr and tNAA/Cr at a TE of 120 ms (0.67 ± 0.09, 1.2 ± 0.04, respectively) relative to 40 ms (0.85 ± 0.04, 1.32 ± 0.02). As expected due to T_1_ and T_2_ relaxation, metabolite ratios calculated from MRSI data acquired at both TEs were different from the real metabolite ratios in the phantom.

### 3.2 Reproducibility/Reliability of the ^1^H MRSI Protocol on Healthy Subjects

Five healthy subjects (3 females, 2 males, aged 29 ± 3.16 years) were included in the study to evaluate reproducibility. SNR, FWHM, and CRLB values for Glu and Gln obtained from scan‐rescan ^1^H sLASER MRSI measurements of healthy subjects at TE of 120 ms are given in Table 1. The mean SNR and FWHM of all spectra were 27.1 ± 3.5 and 0.029 ± 0.009 ppm, respectively. CRLB of Glu and Gln were 3.43% ± 0.56% and 15.1% ± 5.44%, respectively. The lower concentration of Gln compared to Glu in normal brain tissue led to higher CRLB values (fitting uncertainties), as expected. An example of spectral fitting is shown in Figure S1.

**TABLE 1:** Mean and standard deviation of SNR, FWHM, and CRLB values for glutamate (Glu), glutamine (Gln), total N‐acetylaspartate (tNAA), total creatine (tCr), total choline (tCho) and myo‐inositol (mI) spectral fitting obtained from scan‐rescan ^1^H sLASER MRSI measurements of five healthy subjects at TE of 120 ms.

	Session	FWHM (ppm)	SNR	CRLB (%)					
				Glu	Gln	tNAA	tCr	tCho	mI
Subject #1	Scan #1	0.03 ± 0.01	25.2 ± 1.91	3.43 ± 0.50	12.63 ± 1.77	1.00 ± 0.00	1.56 ± 0.005	2.00 ± 0.00	4.87 ± 0.75
	Scan #2	0.03 ± 0.01	25.0 ± 2.04	3.55 ± 0.63	16.28 ± 2.80	1.00 ± 0.00	1.76 ± 0.04	1.98 ± 0.14	5.50 ± 1.12
Subject #2	Scan #1	0.03 ± 0.01	30.5 ± 2.57	3.32 ± 0.47	16.19 ± 5.18	1.00 ± 0.00	1.90 ± 0.03	1.93 ± 0.26	4.70 ± 0.76
	Scan #2	0.03 ± 0.01	34.0 ± 2.86	3.28 ± 0.45	14.35 ± 2.97	1.00 ± 0.00	1.91 ± 0.03	2.00 ± 0.00	4.53 ± 0.64
Subject #3	Scan #1	0.03 ± 0.01	27.5 ± 1.50	3.58 ± 0.62	18.56 ± 7.98	1.00 ± 0.00	1.90 ± 0.03	1.98 ± 0.14	5.60 ± 0.94
	Scan #2	0.03 ± 0.01	27.2 ± 1.89	3.57 ± 0.64	19.44 ± 10.51	1.00 ± 0.00	1.96 ± 0.02	2.00 ± 0.00	5.81 ± 0.86
Subject #4	Scan #1	0.04 ± 0.01	24.2 ± 2.19	3.26 ± 0.46	13.13 ± 1.63	1.00 ± 0.00	1.56 ± 0.05	1.99 ± 0.10	4.87 ± 0.88
	Scan #2	0.03 ± 0.01	26.1 ± 2.25	3.34 ± 0.47	15.46 ± 2.80	1.00 ± 0.00	1.72 ± 0.05	2.00 ± 0.00	4.95 ± 0.83
Subject #5	Scan #1	0.03 ± 0.01	26.0 ± 2.01	3.36 ± 0.48	11.78 ± 1.64	1.00 ± 0.00	1.83 ± 0.04	2.00 ± 0.00	5.60 ± 0.90
	Scan #2	0.02 ± 0.01	25.4 ± 1.48	3.62 ± 0.70	13.13 ± 2.38	1.03 ± 0.01	1.88 ± 0.03	2.00 ± 0.00	6.02 ± 1.11

**FIGURE 3:**
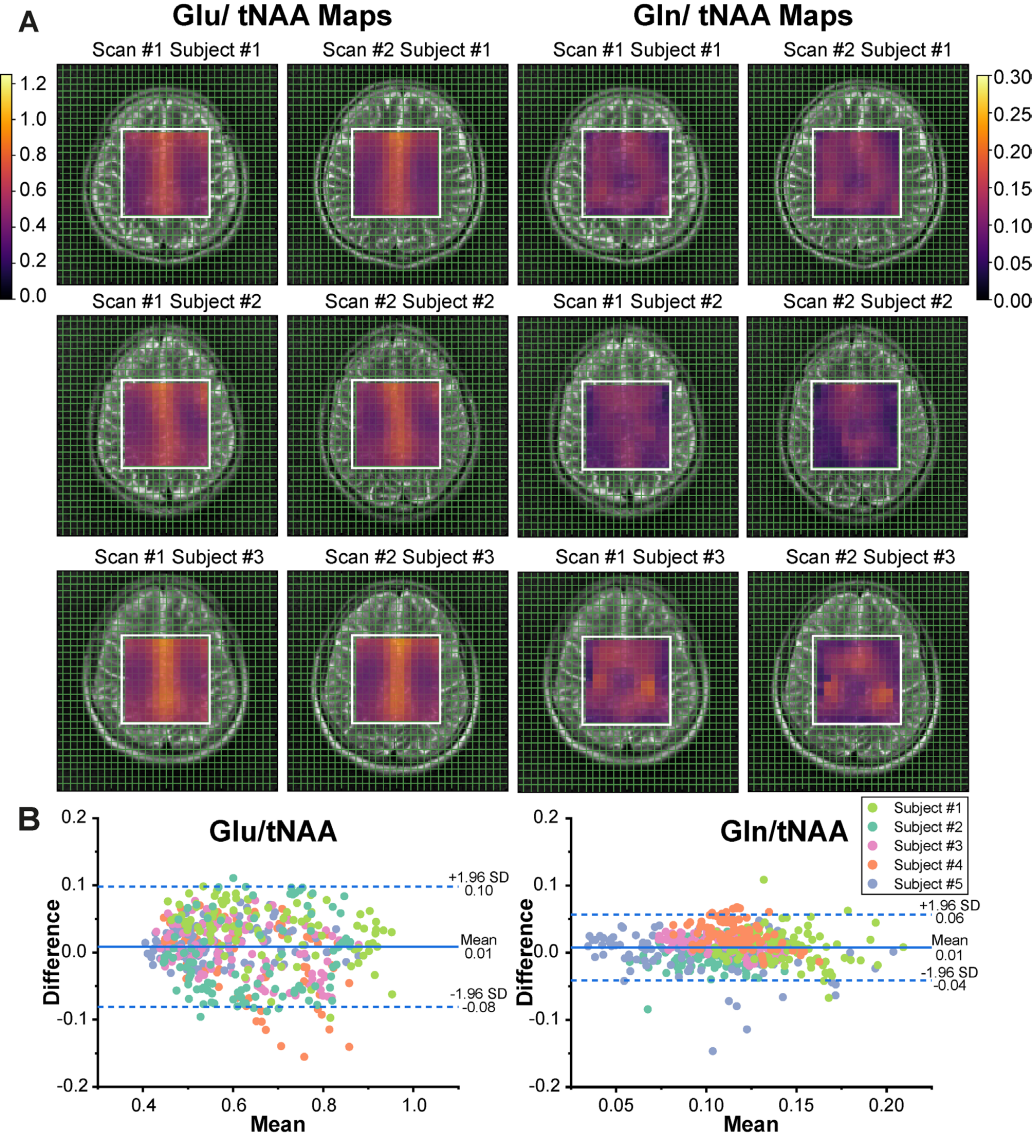
(A) Glutamate/N‐acetylaspartate (Glu/tNAA) (left) and Glutamine/N‐acetylaspartate (Gln/tNAA) (right) maps of three healthy volunteers generated from scan and re‐scan measurements with ^1^H MR spectroscopic imaging using sLASER sequence at 120 ms TE. (B) Bland–Altman plots for Glu/tNAA and Gln/tNAA ratios obtained from the scan‐rescan measurement of five healthy volunteers.

Similar patterns were observed in the Glu/tNAA and Gln/tNAA maps obtained at TE of 120 ms across two repeated sessions (Figure 3A). Bland–Altman plots (difference plots) showed narrow limits of agreement (±1.96 SD) between sessions, with an absolute bias of 0.01 for both Glu/tNAA and Gln/tNAA (Figure 3B).

**FIGURE 4:**
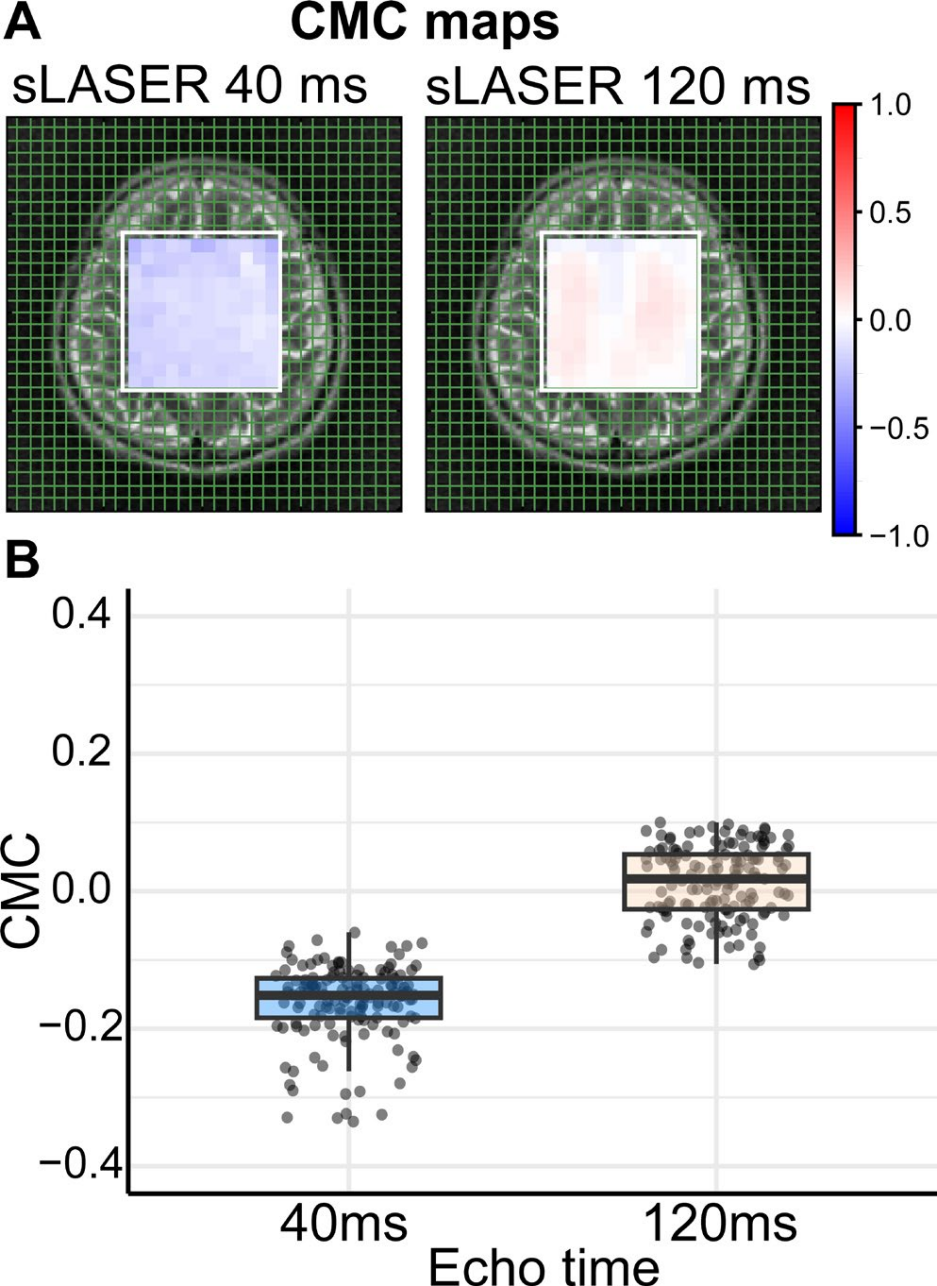
(A) Maps of LCModel coefficients of modeling covariance (CMC) between glutamate (Glu) and glutamine (Gln) for ^1^H MRSI data acquired using sLASER sequence with TE of 40 and 120 ms, registered on T2‐weighted images. CMC map demonstrates a stronger negative correlation (blue contrast) for 40 ms TE than 120 ms TE. (B) Box plots showing the distribution of CMC values for TE of 40 and 120 ms. The data illustrate a shift from predominantly negative values at 40 ms to values more evenly distributed around 0 at 120 ms. Individual data points are overlaid to show variability.

The mean CMC value extracted from data acquired at the conventional short‐TE (40 ms) was −0.16 ± 0.06, indicating a negative association between Glu and Gln (Figure 4). In contrast, at the proposed long‐TE (120 ms), the mean CMC was 0.01 ± 0.05, demonstrating a substantial reduction in the negative association.

### 3.3 Patient Characteristics

Of the 30 patients recruited for the clinical trial, four were excluded as they did not meet the diagnostic criteria of diffuse glioma: One was diagnosed with inflammation, one with a non‐small cell lung cancer metastasis, one with a dysembryoplastic neuroepithelial tumor, and for one patient the indication for biopsy/resection was withdrawn due to non‐tumor diagnosis. All other patients were diagnosed with adult‐type diffuse gliomas, WHO CNS grades 2–4. Following the spectral analysis, two additional patients were excluded from further analysis due to insufficient spectral quality. Tissue samples from the remaining 24 patients were classified as follows: 18 tumors were IDHwt glioblastoma (CNS WHO grade 4 [*n* = 18]), and 6 tumors were IDHmut astrocytoma and oligodendroglioma (CNS WHO grade 2 [*n* = 2], grade 3 [*n* = 4]). To exclude effects originating from the neomorphic enzymatic activity of the mutant IDH protein and to form a uniform cohort, only patients diagnosed with glioblastoma IDHwt were evaluated in this work.

### 3.4 Glutamate and Glutamine Distribution in Glioma

**FIGURE 5:**
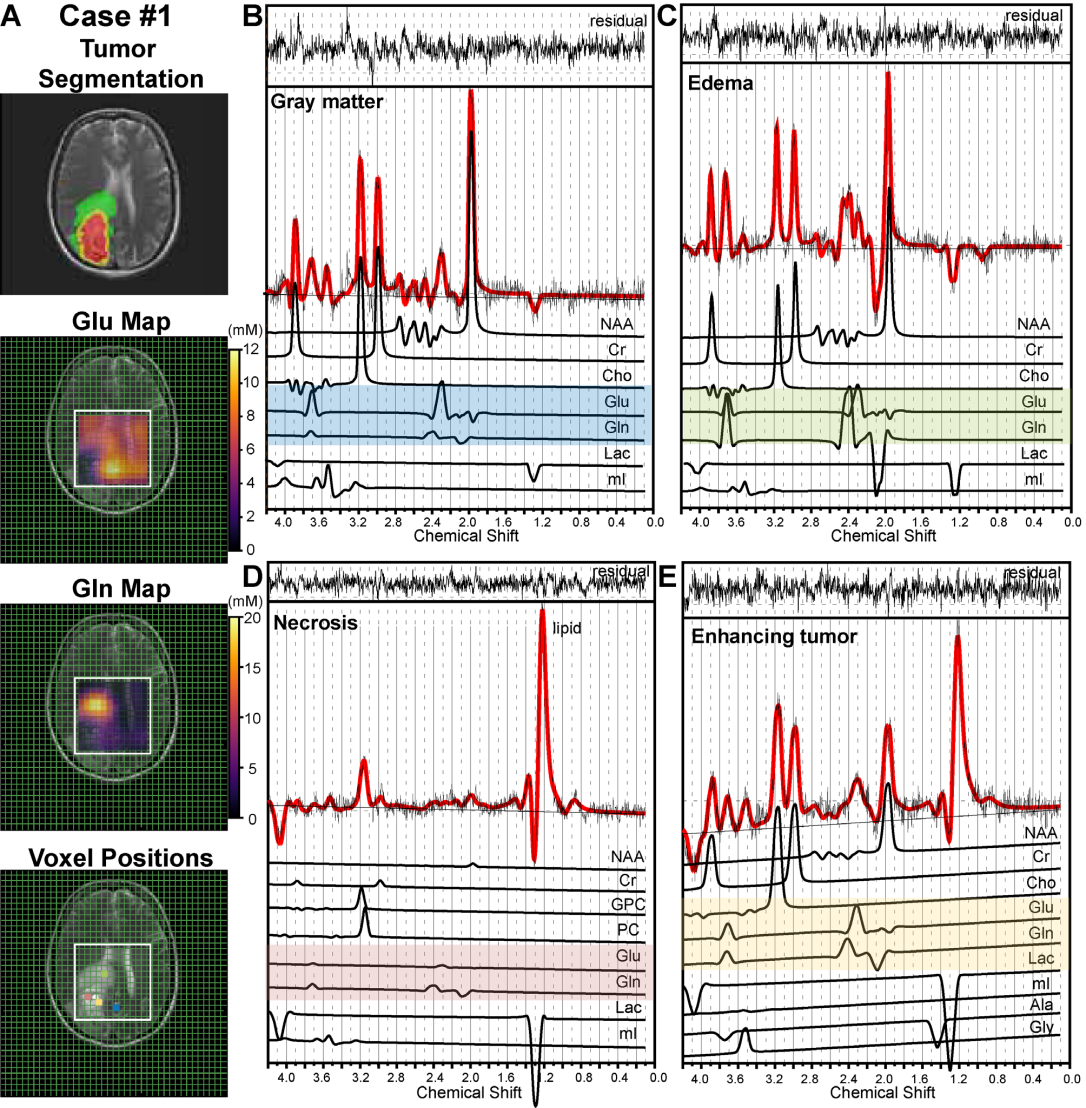
(A) Tumor segmentation, glutamate (Glu) and glutamine (Gln) maps registered on T2‐weighted images of an example patient with IDH wild‐type glioblastoma along with positions of voxels selected from gray matter, edema, necrosis, and enhancing tumor. (B–E) LCModel analysis of the corresponding spectra from selected voxels. The original signal is presented in black, with the LCModel fit overlaid in red. Individual fitting lines generated from simulated metabolite spectra, including baseline correction, are displayed below. Spectral fitting of Glu and Gln is highlighted using colors corresponding to the voxel position, demonstrating their distinguishability at a TE of 120 ms. The residual spectra, presented at the top, were generated by subtracting the experimental spectra from the fitted spectra.

In Figure 5, an example of Glu and Gln distribution in the tumor subregions and contralateral normal‐appearing brain tissue of glioblastoma is shown. Although Gln accumulated in the peritumoral edema, the highest Glu level was found in the gray matter, as expected. The different Glu/Gln ratios for each region, that is gray matter, edema, tumor enhancing, and necrosis, led to distinct spectral patterns in the Glu and Gln region, particularly at the range of 2.0–2.4 ppm, as shown in the spectral fitting.

**FIGURE 6:**
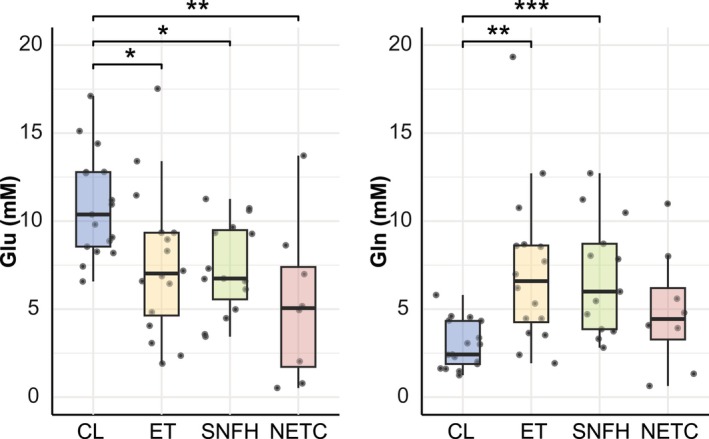
Box plots of glutamate (Glu) and glutamine (Gln) concentrations in tumor subregions, including enhancing tumor (ET), non‐enhancing tumor core (NETC), surrounding non‐enhancing FLAIR hyperintensity (SNFH), and the contralateral (CL) region (mainly WM) in IDH wild‐type glioma. Two data points with the highest Gln levels in SNFH (24.1 and 24.4 mM) were not included in the plot. Results were considered significant at *p < 0.05; **p < 0.01; ***p < 0.001.

In IDHwt glioblastomas, Glu levels were significantly lower across all tumor subregions (NETC: 5.35 ± 4.45 mM, SNFH: 7.39 ± 2.62 mM, and ET: 7.60 ± 4.16 mM) compared to contralateral tissue (10.84 ± 2.94 mM) (Figure 6). Gln levels, on the other hand, were significantly higher in SNFH (9.17 ± 6.84 mM) and ET (7.20 ± 4.42 mM) than in contralateral tissue (2.94 ± 1.35 mM). There was no significant difference in Gln levels between NETC (4.92 ± 3.38 mM) and contralateral tissue (*p* = 0.18). In this study, the contralateral tissue consisted of 72.9% ± 11.2% white matter and 18.8% ± 8.2% gray matter across all subjects.

For the case shown as an example in Figure 5, CMC values were also mapped (Figure S2). The map revealed a mildly heterogeneous distribution of CMC levels across tumor subregions and normal‐appearing contralateral tissue, with a mean of −0.03 ± 0.07, compared to CMC maps obtained from the healthy subject.

## 4 Discussion

In this study, TE for sLASER ^1^H MRSI was optimized to discriminate Glu from Gln at clinical field strengths (3 T). Combining with fully automated multiparametric tumor segmentation, a protocol that allowed us to investigate alterations in glutamatergic mechanisms in tumoral and peritumoral subregions, within a clinically feasible scan time, was developed.

The spectral pattern of Glu and Gln, obtained by J‐modulation at TE of 120 ms, can be exploited to discriminate the signals from both metabolites. Using the ^1^H MRSI vendor‐provided sLASER sequence, Glu and Gln were quantified separately, thereby addressing signal overlap challenges. The approach was validated with phantom measurements, and its reproducibility (scan‐rescan measurements) and reliability (high spectral quality, low and consistent spectral fitting uncertainty) were demonstrated on healthy subjects. Further, the findings showed that the negative association between Glu and Gln spectral fitting at conventional short‐TE was eliminated at the proposed long‐TE, proving improvement in these metabolites' distinguishability.

Another key metabolite that must be accounted for, due to the spectral overlap with Glu and Gln, is 2HG [28], which accumulates in IDHmut glioma tissue as a result of the neomorphic catalytic activity of the mutated enzymes. In the spectral simulations, the specific anti‐phase component of the Gln spectrum shows no spectral overlap with the prominent 2HG peaks, facilitating their distinction. Although this finding is of minor importance in the current study on IDHwt cases, it is highly relevant in a follow‐up study with a focus on IDHmut gliomas.

Another advantage of the proposed protocol is the efficient elimination of baseline modulations originating from cellular macromolecules and lipids. Especially in tumor tissue with the presence of mobile lipids or pathologically altered macromolecules, a reliable baseline modeling is complicated and prone to errors [37, 38]. However, at the long TE suggested here, signals from macromolecules and lipids, which usually have short T_2_ relaxation times, are suppressed, improving the spectral fitting for the quantification of metabolites [31]. Further, some metabolites of interest in tumor metabolism, such as Lac and Ala, exhibit near‐fully inverted, specific spectral patterns at the suggested TE, facilitating their quantification [39, 40].

Although there are previous studies that have optimized TEs to distinguish Glu and Gln spectral patterns with PRESS and STEAM sequences at field strenghts ≤ 3 T, the use of the sLASER sequence offers additional advantages [18, 19]. The STEAM sequence excites only half of the magnetization to generate the stimulated echo, whereas the sLASER sequence utilizes the full magnetization. The PRESS localization suffers from chemical shift displacement errors, which are mitigated in sLASER with the use of high‐bandwidth adiabatic excitation pulses [41]. The sLASER localization scheme also suppresses anomalous J‐evolution, reduces sensitivity to B_1_^+^ inhomogeneities, and prolongs apparent T_2_ relaxation [20]. These advantages are the basis for the recent recommendation of the sLASER sequence by experts' consensus [20].

With the ability to map the spatial distributions of Glu and Gln, the study aimed at monitoring their concentrations in different glioma subregions. Based on the previous experience with mapping metabolite concentrations in brain tumors [39], a fully automated brain tumor segmentation was integrated in the developed protocol [33]. Low Glu levels in all tumor subregions of IDHwt glioma were found compared to contralateral tissue, whereas Gln concentrations were higher in SNFH and ET, but not in NETC. These differences were demonstrated by exemplary spectra from each tumor subregion. In previous studies at field strenghts ≤ 3 T, only the sum of Glu and Gln levels was reported to assess tumor‐specific metabolic alterations due to previously described methodological limitations, hindering their separate assessment [40, 42, 43, 44, 45]. The sometimes contradicting results with regard to Glx levels and their correlations with tumor types and their molecular profiles are likely linked to partial volume effects of single voxel measurements, outdated tumor classifications, and levels of Glu and Gln that alter in different directions, driven by various biological mechanisms [13]. Previous studies have suggested extracellular Glu accumulation in tumor regions [7, 11]. As intracellular and extracellular Glu were measured with most of the signal originating from intracellular Glu, an increase in extracellular concentration could be concealed by the decrease in intracellular Glu. Diffusion‐weighted MRS may offer valuable insights by revealing the compartmental distribution of Glu [46]. The finding of increased Gln levels in IDHwt gliomas aligns with the association between elevated Gln and poor prognosis, as IDHwt itself is a biomarker of poor clinical outcomes [5, 47]. With the emergence of novel drugs targeting glutamatergic and glutaminolysis pathways [12, 14], the developed protocol, which has demonstrated reliability and reproducibility, could be valuable for therapy stratification and monitoring therapy effects in clinical settings within a feasible timeframe.

Recent 7T MRS/I studies on glioma showed distinct maps/levels of Glu and Gln, highlighting the necessity for their separate quantification [48, 49, 50]. However, the reported Glu and Gln levels across these studies are inconsistent. This discrepancy may arise from the use of metabolite ratios, which can obscure changes in real concentrations due to substantial variability in reference metabolites such as tNAA, tCho, and tCr across tumor subregions. Therefore, absolute metabolite quantification along with tumor subregion‐specific evaluations is required for consistently reporting metabolic changes in tumor subregions.

### 4.1 Limitations

One of the limitations of the study is the small sample size (5 healthy subjects and 18 IDHwt glioma cases were included). There are a few assumptions in metabolite quantification. T_1_ and T_2_ relaxation corrections of metabolite signals were performed using literature values for healthy brain tissue, as relaxation times of metabolites in brain tumors are not consistently reported in the literature. Additionally, the calibration in the quantification technique relies on the water signal obtained from contralateral normal‐appearing white matter, with corrections for relaxation and water content based on non‐age‐specific literature values, as detailed in the Supporting Information.

Although tumor segmentation was integrated into the ^1^H MRSI analysis to carefully evaluate metabolite changes across tumor subregions, the voxel size of 6 × 6 × 12 mm^3^ may still cause partial volume effects, preventing the detection of true values for changes in specific regions. In this study, voxels were assigned to a tumor subregion with a lower threshold of 50% partial volume. Although MRSI with smaller voxels would be preferred, the acquisition results in lower signal amplitude and requires longer acquisition times, limiting the reliability and feasibility of the technique in clinical settings.

### 4.2 Conclusion

A distinctive MR spectroscopy‐based in vivo analysis of Glu and Gln in glioma tumor and peritumoral regions will offer important insight into tumor biology and metabolic subtypes. Here, a long‐TE (120 ms) sLASER ^1^H MRSI protocol combined with a multiparametric brain tumor segmentation was employed and validated to measure Gln and Glu concentrations in glioma patients. This protocol can help guide patient stratification in the development of novel metabolic drugs and further our understanding of the glioma microenvironment.

## Conflicts of Interest

M.W.R. has received research funding from UCB as well as honoraria for advisory board participation from Alexion and Servier. J.P.S. has received honoraria for consulting or advisory board membership as well as travel or accommodation support from Abbvie, Medac, Novocure, Roche, Servier and UCB. All other authors declare no conflicts of interest.

## Supporting information


Data S1.

## Data Availability

The data that support the findings of this study are available from the corresponding author upon reasonable request.
