# Supplementary material for: Optimized Long‐TE 1H sLASER MR Spectroscopic Imaging at 3T for Separate Quantification of Glutamate and Glutamine in Glioma
Source: J Magn Reson Imaging. 2025 Apr 8;62(3):890–901. doi: 10.1002/jmri.29787 (PMC12335340; doi:10.1002/jmri.29787)
Supplement: Supplementary file 1 — Data S1. [file JMRI-62-890-s001.pdf]

Supplementary Information: Optimized Long-TE ^{1}H sLASER MR Spectroscopic Imaging at 3T for Separate Quantification of Glutamate and Glutamine in Glioma

MRSI sequence protocol

Supplementary Table 1. MRSI sequence protocol. A 20-channel ^{1}H head coil was used to record a 2D T2-weighted TSE sequence in the axial plane (2.5 min), a 3D T1-weighted GRE sequence (4 min), and a 2D ^{1}H CSI with sLASER localization at TE 120 ms and additional lower resolution water reference.

Pulse<br>Sequence	2D ^{1}H sLASER MRSI<br>(for healthy volunteer measurements)	2D ^{1}H sLASER MRSI<br>(for patient measurements)	2D ^{1}H sLASER MRSI (water reference)<br>(for patient measurements)
TE	120 ms	120 ms	120 ms
TR	2500 ms	2000 ms	2000 ms
Flip Angle	90°	90°	90°
Slice Thickness	20 mm	12 mm	12 mm
Matrix Size and Field of View	16 x 16 at 200 × 200 mm^2 interpolated to 32 x 32	20 x 20 at 240 × 240 mm^2 interpolated to 40 x 40	10 x 10 at 240 × 240 mm^2 interpolated to 20 x 20
Voxel size	6.25 x 6.25 x 20 mm^3	6 x 6 x 12 mm^3	12 x 12 x 12 mm^3
Vector size	2048	2048	2048
Bandwidth	1200 Hz	2000 Hz	2000 Hz
Acquisition time	10:01 m	10:52 m	1:46 m
Number of Averages	3	2	1
Water Suppression	CHESS	CHESS	None

Spectral Fitting

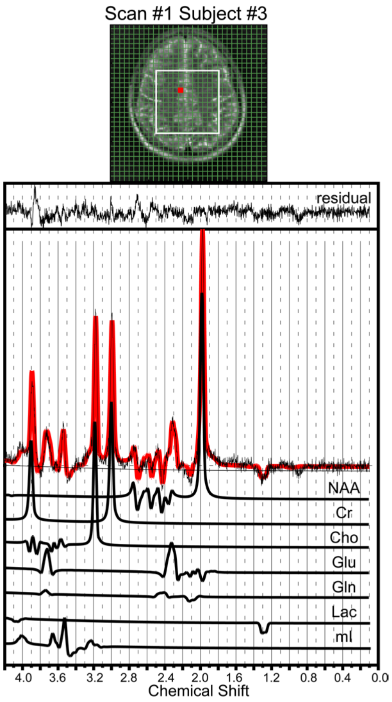

Supplementary Figure 1. (A) An example analysis of ^{1}H long-TE (120 ms) sLASER MRSI data using LCModel with simulated basis set. Red box indicates voxel positioning on T2-weighted image. The original signal is presented in black, and the LCModel fit in red. The individual fitting lines of prominent metabolites are shown below. NAA indicates N-acetylaspartarte; Cr, creatine; Cho, choline; Glu, glutamate; Gln, glutamine; Lac, lactate; mI, myo-inositol.

Quantification of ^{1}H-MRS detectable Metabolites

Following 2D ^{1}H sLASER MRSI measurement at 120 ms with 20x20 nominal matrix size, a lower resolution (10x10 matrix size) sLASER MRSI sequence was employed without performing water suppression to acquire water signals for metabolite quantification. Both MRSI data were interpolated to 40x40 matrix size. The poor point spread function (PSF) of the water reference MRSI data was matched with the PSF of tissue segmentation for normal-appearing brain tissue by reducing its resolution using an appropriate hamming filter. Metabolite and water concentrations in tissue were calculated as follows[1,2]:

$$C_{met}\left(\frac{mol}{L}\right) = \frac{mol_{met}}{V_{tissue}}, C_W\left(\frac{mol}{L}\right) = \frac{mol_W}{V_{tissue}}, \qquad (1)$$

where mol_{met} represents moles of metabolites and mol_w, moles of water in tissue. Here tissue volume (V_{tissue}) was assumed to be equal to the voxel volume. When MR signals are acquired with full T_1 relaxation and no T_2 relaxation (TE=0), the relationship between metabolite (S_{met}) and water (S_w) signal intensity can be described as follows:

$$\frac{S_{met}}{S_W} = \frac{N_{met} \times mol_{met}}{N_W \times mol_W}, \qquad (2)$$

where N_{met} and N_W represent the number of metabolite and water protons (i.e., $N_W = 2$) contributing to S_{met} and S_W. Using the equations above, C_{met} can be calculated as follows:

$$C_{met} = \frac{S_{met} \times N_w}{S_W \times N_{met}} \times C_W. \qquad (3)$$

Concentration of water in the voxel (C_W) can be calculated based on GM, WM and CSF segmentation created employing SPM12 (version 7771)[3] as follows:

$$C_W = (f_{WM} \times C_{H2O\_WM} + f_{GM} \times C_{H2O\_GM} + f_{CSF} \times C_{H2O\_CSF}), \qquad (4)$$

where f_{WM}, f_{GM} and f_{CSF} represent fractions of WM, GM, CSF in the voxel. C_{H2O_WM}, C_{H2O_GM} and C_{H2O_CSF} are concentration of water in each tissue type which can be calculated as follows:

$$C_{H2O_{WM}} = C_{H2O} \times f_{w\_WM};\ C_{H2O\_GM} = C_{H2O} \times f_{w\_GM};\ C_{H2O\_CSF} = C_{H2O} \times f_{w\_CSF}, \qquad (5)$$

where C_{H2O} is pure water concentration (55.56 M). f_{w_WM}, f_{w_GM}, and f_{w_CSF} represent the fraction of water content in each tissue type and used literature values are 0.65, 0.78, and 0.97, respectively.

To correct water signal intensities acquired from contralateral normal-appearing brain tissue, relaxation correction factor for each tissue fraction (r_{WM}, r_{GM}, r_{CSF}) was calculated using literature values for T_1 ($T_{1_{WM}}, T_{1_{GM}}, T_{1_{CSF}}$) and T_2 ($T_{2_{WM}}, T_{2_{GM}}, T_{2_{CSF}}$) relaxation as follows:

$$r_{WM} = \frac{e^{\frac{TE}{T_{2WM}}}}{1-e^{\frac{-TR}{T_{1WM}}}};\ r_{GM} = \frac{e^{\frac{TE}{T_{2GM}}}}{1-e^{\frac{-TR}{T_{1GM}}}};\ r_{CSF} = \frac{e^{\frac{TE}{T_{2CSF}}}}{1-e^{\frac{-TR}{T_{1CSF}}}}, \qquad (6)$$

$$S_{W\_r} = S_W \times \left(\frac{f_{WM} \times C_{H2O_{WM}} \times r_{WM} + f_{GM} \times C_{H2O_{GM}} \times r_{GM} + f_{CSF} \times C_{H2O_{CSF}} \times r_{CSF}}{C_w}\right), \qquad (7)$$

where S_{W_r} represents partially T_1 and T_2 relaxed water signal intensity acquired with sequence parameters of echo time (TE) and repetition time (TR). After extracting S_W using equation above, the last term which must be calculated in Eq. 3 is the relaxation time corrected signal intensity of the reference metabolite (S_{met}) and it can be calculated as follows:

$$r_{met} = \frac{e^{\frac{TE}{T_{2met}}}}{1-e^{\frac{-TR}{T_{1met}}}}, \qquad (8)$$

$$S_{met\_r} = S_{met} \times r_{met}, \qquad (9)$$

where S_{met_r} represents partially T_1 and T_2 relaxed metabolite signal intensity acquired with sequence parameters of echo time (TE) and repetition time (TR). Now, we have all the terms to calculate C_{met} using Eq. 3. After performing this calculation for four calibration voxels in normal-appearing brain tissue, the relationship between relaxation corrected signal intensity of reference metabolite ($S_{met}(CL_{ref})$) and its concentration ($C_{met}(CL_{ref})$) is generalized to all MRSI voxels (i,j). This approach is based on minimal effects of B1+/- inhomogeneity, since the sLASER sequence with high-bandwidth adiabatic radiofrequency pulses is less sensitive to B1+ inhomogeneities[4], while the "prescan normalize" option in the MRSI sequence was used to reduce B1- inhomogeneities. Thus, the calculation of the metabolite concentration ($C_{met}(i,j)$) in each voxel using relaxation corrected signal intensity of metabolite of interest ($S_{met}(i,j)$) can be written as follows:

$$C_{met}(i,j) = \frac{S_{met}(i,j)}{S_{met}(CL_{ref})} \times C_{met}(CL_{ref}). \qquad (10)$$

MRSinMRS checklist

The Minimum Reporting Standards for in vivo Magnetic Resonance Spectroscopy (MRSinMRS) checklist[5] can be found in Supplementary Table 2.

Supplementary Table 2. MRSinMRS checklist for our ^1H MRSI protocol.

1. Hardware		
a. Field strength [T]	3 T	3 T
b. Manufacturer	Siemens	Siemens
c. Model (software version if available)	Prisma (VE11C)	Prisma (VE11C)
d. RF coils: nuclei (transmit/ receive), number of channels, type, body part	20 ch ^1H head coil	20 ch ^1H head coil
e. Additional hardware	N/A	N/A
2. Acquisition		
a. Pulse sequence	2D ^1H Semi-LASER CSI (vendor-based)	2D ^1H Semi-LASER CSI (vendor-based) - **water reference for patient measurements**
b. Volume of Interest (VOI) locations	For healthy volunteer measurements: medial frontoparietal<br><br>For patient measurements: tumor and contralateral	For patient measurements: tumor and contralateral
c. Nominal VOI size [cm^3, mm^3]	For healthy volunteer measurements: 80*80*20 mm^3<br><br>For patient measurements: adjusted according to tumor volume with a slice thickness of 12 mm for patients.	For patient measurements: adjusted according to tumor volume with a slice thickness of 12 mm for patients.

d. Repetition Time (TR), Echo Time (TE) [ms, s]	For healthy volunteer measurements: TR = 2500 ms, TE = 120 ms<br><br>One volunteer measured with TE = 40 ms for comparison<br><br>For patient measurements: TR = 2000 ms, TE = 120 ms	For patient measurements: TR = 2000 ms, TE = 120 ms
e. Total number of Excitations or acquisitions per spectrum<br><br>In time series for kinetic studies<br><br>i. Number of Averaged spectra (NA) per time-point<br>ii. Averaging method (e.g. block-wise or moving average)<br>iii. Total number of spectra (acquired / in time-series)	For healthy volunteer measurements: 3<br><br>For patient measurements: 2	For patient measurements: 1
f. Additional sequence parameters<br><br>(spectral width in Hz, number of spectral points, frequency offsets)<br><br>If STEAM:, Mixing Time (TM)<br><br>If MRSI: 2D or 3D, FOV in all directions, matrix size, acceleration factors, sampling method	For healthy volunteer measurements: sampling; 1200 Hz, 2048 points<br><br>delta frequency = −2.7 ppm<br><br>flip angle = 90°<br><br>2D: 200 × 200 × 20 mm^3 FOV; matrix size 16 x 16 interpolated to 32 x 32; no acceleration factor; weighted distribution<br><br>For patient measurements: sampling; 2000 Hz, 2048 points<br><br>delta frequency = −2.7 ppm<br><br>flip angle = 90°	For patient measurements: sampling; 2000 Hz, 2048 points<br><br>delta frequency = 0 ppm<br><br>flip angle = 90°<br><br>2D: 240 × 240 × 12 mm^3 FOV; matrix size 10 x 10 interpolated to 20 x 20; no acceleration factor

	2D: 240 × 240 × 12 mm^3 FOV; matrix size 20 x 20 interpolated to 40 x 40; no acceleration factor; weighted distribution sampling;	
g. Water Suppression Method	CHESS	None
h. Shimming Method, reference peak, and thresholds for "acceptance of shim" chosen	Automated 3D B0 field mapping technique (GRE-SHIM for brain)	Automated 3D B0 field mapping technique (GRE-SHIM for brain)
i. Triggering or motion correction method<br><br>(respiratory, peripheral, cardiac triggering, incl. device used and delays)	N/A	N/A
3. Data analysis methods and outputs		
a. Analysis software	LCmodel 6.2	LCmodel 6.2
b. Processing steps deviating from quoted reference or product	Basis set created using jMRUI 6.0 plug-in NMR-ScopeB	Basis set created using jMRUI 6.0 plug-in NMR-ScopeB
c. Output measure<br><br>(e.g. absolute concentration, institutional units, ratio)Processing steps deviating from quoted reference or product	For healthy volunteer measurements: Ratios to tNAA<br><br>For patient measurements: Ratios to water	Used as water reference
d. Quantification references and assumptions, fitting model assumptions	The basis set included spectra of N-acetylaspartate (NAA), N-acetylaspartylglutamate (NAAG), choline (Cho), glycerophosphocholine (GPC), creatine (Cr),	

	glutamate (Glu), glutamine (Gln), myo-inositol (mI), lactate (Lac), γ-aminobutyric acid (GABA), glutathione (GSH), glycine (Gly), alanine (Ala), glucose (Glc), valine (Val). The spline function in LCModel which models a baseline composed of macromolecules and lipid signals was used applying the control parameter DKNTMN (minimum allowed spacing between spline knots) of 5 ppm.	
4. Data Quality		
a. Reported variables<br><br>(SNR, Linewidth (with reference peaks))	SNR and linewidths are presented in Table 1 for healthy spectra.	NA
b. Data exclusion criteria	LCModel SNR < 3, LCModel FWHM > 0.1 ppm, existing artifacts	NA
c. Quality measures of postprocessing Model fitting (e.g. CRLB, goodness of fit, SD of residual)	CRLB < 10% for total choline.	NA
d. Sample Spectrum	Supplementary Figure 1 (healthy volunteer), Figure 3 (patients)	NA

LCModel Coefficient of Modeling Covariance Maps for Brain Tumor MRSI Dataset

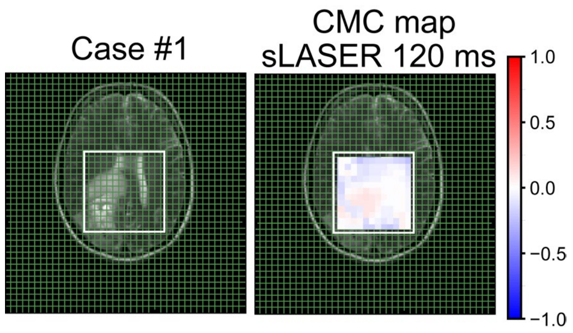

Supplementary Figure 2. Map of LCModel coefficients of modeling covariance (CMC) between glutamate and glutamine for ^{1}H MRSI data acquired from an IDHwt glioma patient using sLASER sequence with TE of 120 ms, registered on T2-weighted image.

Spectral Simulations of Glutamate, Glutamine, and 2-Hydroxyglutarate

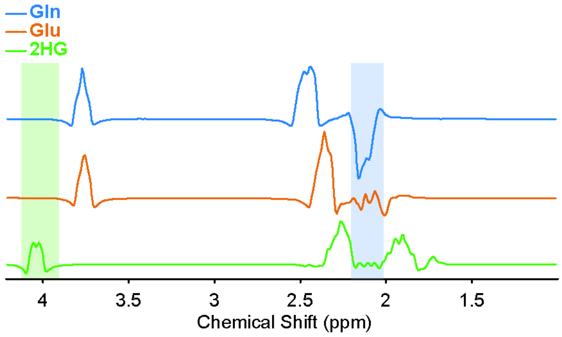

Supplementary Figure 3. Simulated spectra of glutamate (Glu), glutamine (Gln) and 2-hydroxyglutarate (2HG) at 120 ms TE for ^{1}H MRS using sLASER sequence. No clear 2HG peaks were observed within the spectral range corresponding to the anti-phase component of the Gln spectrum (highlighted with a blue box). In addition, at the 3.5 - 4.1 ppm spectral range, peaks of Glu and Gln appear at ~3.75 ppm and a peak of 2HG at ~4.02 ppm.

References

1. Near J, Harris AD, Juchem C, et al. Preprocessing, analysis and quantification in single-voxel magnetic resonance spectroscopy: Experts' consensus recommendations. *NMR Biomed* 2021;34:e4257.

2. Gasparovic C, Song T, Devier D, et al. Use of tissue water as a concentration reference for proton spectroscopic imaging. *Magn Reson Med* 2006;55:1219–26.

3. Friston KJ, ed. *Statistical parametric mapping: the analysis of funtional brain images*. 1st ed. Amsterdam ; Boston: Elsevier/Academic Press; 2007.

4. Öz G, Deelchand DK, Wijnen JP, et al. Advanced single voxel ^{1} H magnetic resonance spectroscopy techniques in humans: Experts' consensus recommendations. *NMR in Biomedicine* 2021;34.

5. Lin A, Andronesi O, Bogner W, et al. Minimum Reporting Standards for in vivo Magnetic Resonance Spectroscopy (MRSinMRS): Experts' consensus recommendations. *NMR in Biomedicine* 2021;34.
